# Meteorin Regulates Mesendoderm Development by Enhancing Nodal Expression

**DOI:** 10.1371/journal.pone.0088811

**Published:** 2014-02-18

**Authors:** Yoon-Young Kim, Jin-Sook Moon, Min-chul Kwon, Juhee Shin, Sun-Kyoung Im, Hyun-A Kim, Jin-Kwan Han, Young-Yun Kong

**Affiliations:** 1 School of Biological Sciences, College of Natural Sciences, Seoul National University, Seoul, South Korea; 2 Division of Molecular and Life Science, Department of Life Sciences, POSTECH, Pohang, South Korea; Laboratoire de Biologie du Développement de Villefranche-sur-Mer, France

## Abstract

During gastrulation, distinct lineage specification into three germ layers, the mesoderm, endoderm and ectoderm, occurs through an elaborate harmony between signaling molecules along the embryonic proximo-distal and anterior-posterior axes, and Nodal signaling plays a key role in the early embryonic development governing embryonic axis formation, mesoderm and endoderm specification, and left-right asymmetry determination. However, the mechanism by which Nodal expression is regulated is largely unknown. Here, we show that Meteorin regulates Nodal expression and is required for mesendoderm development. It is highly expressed in the inner cell mass of blastocysts and further in the epiblast and extra-embryonic ectoderm during gastrulation. Genetic ablation of the *Meteorin* gene resulted in early embryonic lethality, presumably due to impaired lineage allocation and subsequent cell accumulation. Embryoid body culture using Meteorin-null embryonic stem (ES) cells showed reduced *Nodal* expression and concomitant impairment of mesendoderm specification. Meteorin-null embryos displayed reduced levels of *Nodal* transcripts before the gastrulation stage, and impaired expression of *Goosecoid*, a definitive endoderm marker, during gastrulation, while the proximo-distal and anterior-posterior axes and primitive streak formation were preserved. Our results show that Meteorin is a novel regulator of *Nodal* transcription and is required to maintain sufficient Nodal levels for endoderm formation, thereby providing new insights in the regulation of mesendoderm allocation.

## Introduction

During embryonic axis formation, lineage specification is governed by the reciprocal signaling including Wnt, Bone morphogenetic protein (BMP), and Nodal signaling pathways among epiblast, extra-embryonic ectoderm, and visceral endoderm cell populations [1], [2], [3], [4], [5], [6], [7], [8]. The proximo-distal (P–D) axis in the epiblast is primarily established by Nodal signaling that activates its antagonists *Lefty1* and *Cerberus-like* (*Cerl*) in the distal region of the visceral endoderm [9], [10]. Activation of these antagonists in the distal visceral endoderm restricts Nodal target gene expression to the proximal region. The movement of the distal visceral endoderm toward the anterior side of the conceptus further generates the anterior-posterior (A–P) axis [5], [11]. The differential gene expression patterns of several signaling pathways along the P–D and A–P axes subsequently guide proper embryonic pattern formation and successful embryogenesis [5], [12]. Although the significance of embryonic axes formation is well documented in genetic studies, the mechanisms by which these embryonic axes cause the emergence of further lineage specification remain unclear.

Nodal, a member of the Transforming growth factor-beta (TGF-β) superfamily [1], binds type I and II activin receptors and the co-receptor Cripto, which leads to phosphorylation of Smad2/3. Phosphorylated Smad2/3 interacts with Smad4, translocates to the nucleus, and acts as a transcriptional activator with several regulatory factors such as Forkhead box protein H1 (FoxH1) [13]. This Nodal signaling plays a key role in the early embryonic development governing P–D and A–P axes formation [5], [12], [14], endoderm and mesoderm specification [7], [15], [16], and left-right asymmetry determination [17], through positive regulation of its own expression as well as that of Lefty2, a negative regulator of Nodal signaling [18], [19]. Although Nodal plays a critical role in early pattern formation, a more in-depth understanding of the regulation of Nodal expression has yet to be obtained.

Meteorin was initially identified as a secreted protein. During embryonic neurogenesis, it is expressed in neural progenitors and astrocyte precursors, and may play roles in glial cell differentiation and axonal network formation [20]. Jorgensen *et al.* reported that Meteorin is also broadly expressed in astrocytes, Bergmann glial cells, and some interneurons in the adult brain, suggesting that it may be implicated in gliogenesis and angiogenesis [21], [22]. Moreover, recent studies suggested that it has a neurotrophic potential in several *in vivo* injury models [23], [24]. However, its physiological roles in neurogenesis, gliogenesis, and angiogenesis have not yet been determined through genetic models yet. In this study, we disrupted the *Meteorin* gene by homologous recombination. Unexpectedly, we found that *Meteorin^−/−^* mice show early embryonic lethality. Consistent with this, *Meteorin* was highly expressed during the preimplantation and gastrulation stages, suggesting that Meteorin may play a role in early embryogenesis. Experiments using cultured embryoid bodies (EBs), which recapitulate early embryonic developmental processes [25], revealed that the levels of Nodal and its downstream target genes, *Lefty1* and *Cerl*, were decreased in *Meteorin^−/Δ^* EB cultures. Reduced expression of Nodal target genes was rescued by exogenous Nodal recombinant protein, suggesting that Nodal signaling itself is intact in the *Meteorin^−/Δ^* cells. Reduced levels of Nodal signaling and subsequent impairment in mesendoderm allocation were consistently observed upon disruption of *Meteorin* in both *in vivo* and *in vitro* systems, in agreement with the previous concept that graded Nodal signaling governs cell fate decisions during gastrulation [7], [26]. Our data show that regulation of Nodal signaling by Meteorin is required for mouse embryonic development.

## Materials and Methods

### Ethics statement

All the mice used in this study were maintained in the specific pathogen-free facility of Seoul National University, and the experiments followed the guidelines of the animal ethics committee. All animal experiments were approved by the Seoul National University Institutional Animal Care and Use Committee (Approval number: SNU120501-9).

### Generation of *Meteorin*-knockout mice

Part of *Meteorin* exon 4 and its 3′ downstream sequence were substituted with an IRES-LacZ-Puro cassette, and the *diphtheria toxin A* (DTA) gene was introduced for negative selection. After electroporation of E14Tg2A embryonic stem (ES) cells with the targeting vector, clones were selected by puromycin treatment. Several clones were verified by Southern blot with a flanking probe (Fig. S1), and a number of positive clones were injected into C57BL/6J blastocysts to generate chimeras. After germline transmission, *Meteorin^+/−(puro)^* mice were bred with Protamine-Flpe mice for excision of the flippase recognition target (FRT)-flanked puromycin resistance gene. In this study, more than 6 rounds of breeding were carried out with C57BL/6J mice to obtain congenic mice.

### Generation of *Meteorin^−/Δ^*ES cells

DNA fragments containing the third and fourth exons of the *Meteorin* gene were flanked by loxP sequences in the targeting vector, and the neomycin resistance gene flanked by FRT sequences was introduced between the loxP sequences. The DTA gene was also inserted in the vector for negative selection. E14Tg2A ES cells were sequentially electroporated with targeting vectors. After neomycin and puromycin double selection, one ES cell line was identified by Southern blot screening. Cre expression was induced by transient expression of the pCAGGS-Cre vector, and the resulting *Meteorin^−/Δ^* cells were selected for further experiments (see Fig. S2). The pCAGGS-Cre and pCAGGS-mock vector were kindly provided by Ken-ichi Yamamura (Kumamoto University, Japan).

### Embryo preparation, section, and hematoxylin and eosin (H&E) staining

Embryos at each developmental stage were collected from timed-pregnant mice. The noon of the day of plug detection was designated as E0.5. For histological sectioning, the embryos were obtained in ice-cold PBS and fixed in 4% paraformaldehyde at 4°C overnight. After the samples were washed with distilled water overnight, they were subjected to serial dehydration steps and paraffin embedding. Four-micrometer-thick sections were cut for staining with hematoxylin and eosin. All images were captured with a Zeiss Axio Imager A2 microscope and Olympus DP70 camera or Diagnostic SPOTFlex camera.

### Whole-mount *in situ* hybridization

Whole-mount *in situ* hybridization was performed as described previously [27]. Gene-specific riboprobe vectors were generated by cloning a nucleotide fragment of each gene into the pGEM-T Easy vector. The sequences of the primers used to amplify the cDNA fragments have been provided in Table S1. The *Meteorin, Lefty1/2, Cerl, Sox17*, and *BMP4* probes were kindly provided by Kyu-Won Kim (Seoul National University, South Korea), Hiroshi Hamada (Osaka University, Japan), José António Belo (University of Algarve, Portugal), Yoshiakira Kanai (The University of Tokyo, Japan) and Paul Overbeek (Baylor College of Medicine, USA), respectively. All images were captured with a Leica MZ16 and Olympus DP70 camera or Diagnostic SPOTFlex camera.

### ES cell culture and EB culture

ES cells were maintained in ES cell media supplemented with or without 300 µg/ml G418 or 3 µg/ml puromycin on mitomycin C-treated mouse embryonic fibroblasts. Recombinant mouse Nodal (rmNodal; R&D, USA) or recombinant mouse Meteorin (kindly provided from Dr. Kyu-Won Kim; Seoul National University, South Korea) were applied to the culture medium to rescue the defects of Meteorin-deficient ES cells. *Meteorin* cDNA was cloned into the pCAGGS vector (pCAGGS-m*Meteorin*), and *Meteorin^−/Δ^* ES cells were transfected using Lipofectamine™ 2000 reagent (Invitrogen, USA) to induce the transient expression of Meteorin. EBs were cultured as previously described [28].

### qRT-PCR analysis

Total RNA isolation, reverse transcription, and real-time qRT-PCR were conducted as previously described [29]. The sequences of the primers used in this study have been provided in Table S1. P-values were calculated using the Student's t-test.

### Western blots

For western blot assays, cells were harvested and sonicated in IP buffer (1% Triton X-100, 80 mM NaCl, 3 mM EDTA, and 50 mM HEPES with protease inhibitors). Generally, 5∼20 µg of protein from lysate supernatants was separated by size, blotted with each primary and secondary antibody, and detected with WEST-ZOL plus (Intron). Rabbit anti-β-actin (Sigma), rabbit anti-p-Smad2 (Cell Signaling, #3101), rabbit anti-Smad2 (Cell Signaling, #5339), and rabbit anti-p-Smad2 (Cell Signaling, #3101) were used for this research.

## Results

### *Meteorin* expression in early embryos

**Figure 1:**
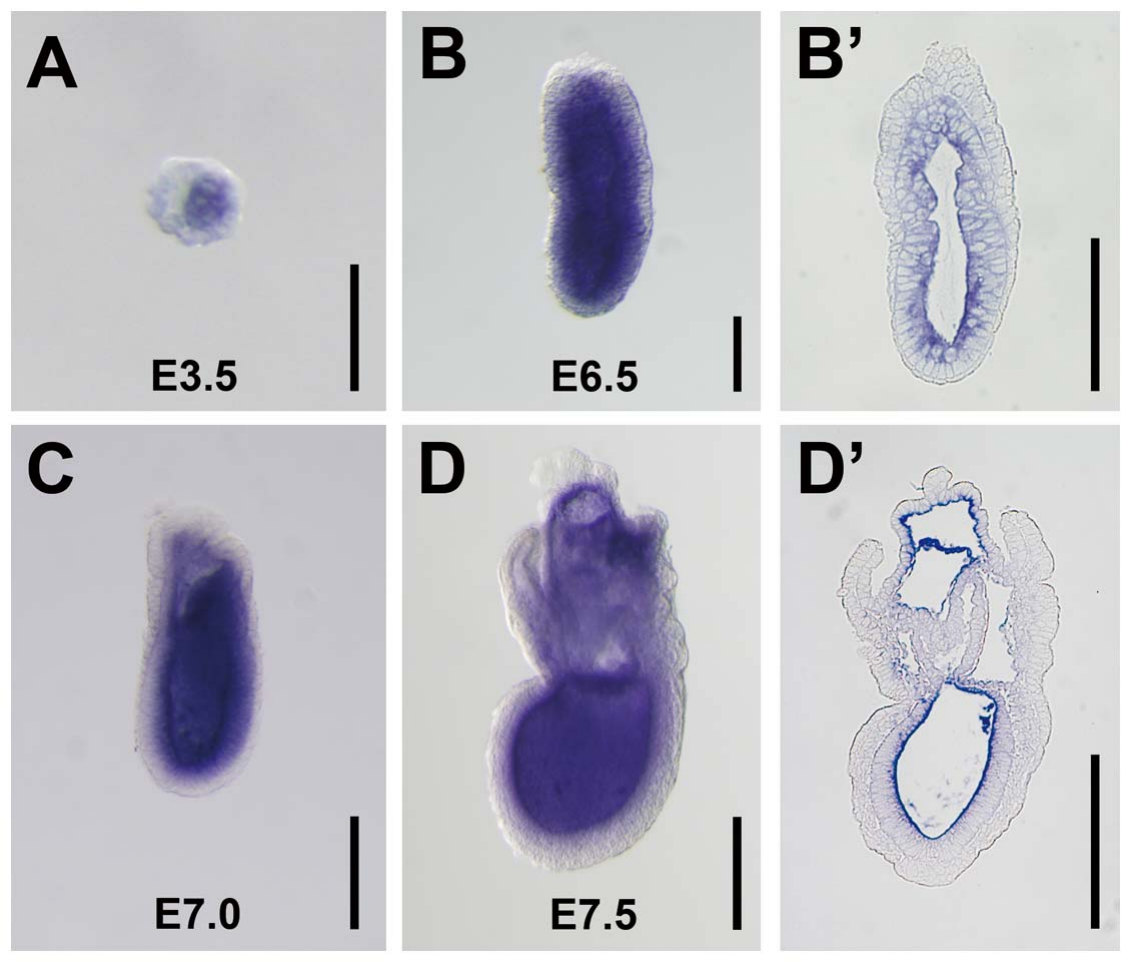
Expression of *Meteorin* in early embryogenesis. **(A–D)** Expression of *Meteorin* mRNA at different stages of development was analyzed by whole-mount *in situ* hybridization with embryos from a timed-pregnant mouse. **(A)** *Meteorin* was expressed in the inner cell mass at the E3.5 blastocyst stage. **(B, B′)** At E6.5, the pre-steak stage, *Meteorin* was expressed in the extra-embryonic ectoderm and epiblast, but not in the visceral endoderm. **(C, D, D′)** During E7.0–E7.5, the mid- to late-gastrulation stages, Meteorin expression was sustained in extra-embryonic ectoderm and the epiblast, but not in extra-embryonic tissue. **(B′ and D′)** After *in situ* hybridization with a probe specific for *Meteorin* at each developmental stage, embryos were fixed, embedded in paraffin, and then sectioned sagittally. The expression level was relatively high in cells close to the proamniotic cavity. All experiments were conducted with more than 5 embryos at each developmental stage and the representative images were captured. Scale bars in A, B, and B′: 100 µm. Scale bars in C, D, D′: 200 µm.

To determine whether Meteorin is expressed earlier than reported [20], we analyzed mRNA expression of *Meteorin* in early embryos through *in situ* hybridization. We found that *Meteorin* was expressed as early as the blastocyst stage and that its expression was restricted to the inner cell mass (Fig. 1A). After implantation, it was ubiquitously expressed throughout epiblast and extra-embryonic ectoderm cells, but not in the visceral endoderm at E6.5 (Fig. 1B, B′) and was maintained in these areas as embryonic development progressed. It was highly expressed in the epiblast close to the proamniotic cavity with a gradient pattern and vague in the outer epiblast adjacent to visceral endoderm (Fig. 1C, D, and D′).

### Impaired gastrulation and early embryonic lethality in Meteorin-null embryos

To elucidate the biological function of Meteorin in early embryonic development, we generated Meteorin-null mice using a gene-targeting method. *Meteorin^+/−^* mice were viable and fertile with no apparent differences compared to wild-type mice, but no viable *Meteorin^−/−^* mice were observed from heterozygous matings at birth, suggesting that Meteorin deficiency results in embryonic lethality. To determine when *Meteorin^−/−^* embryos died, embryos from timed-pregnant heterozygous females at differential gestational stages were genotyped. At earlier than E7.0, *Meteorin^−/−^* embryos appeared in the expected Mendelian ratio. At E7.5, several *Meteorin^−/−^* embryos were obtained but their proportions were beyond the Mendelian ratio. At E8.5, no *Meteorin^−/−^* embryos were observed, indicating that *Meteorin^−/−^* embryos die between E7.0 and E8.5 (Table 1).

**Table 1:** Early embryonic lethality in *Meteorin^−/−^* embryos.

	Number of embryos for each genotype (%)			
Age of embryos	*Meteorin^+/+^*	*Meteorin^+/−^*	*Meteorin^−/−^*	Total n
E8.5	13 (33.33)	26 (66.67)	0 (0)**^A^**	39
E7.5	20 (35.71)	30 (53.57)	6 (10.71)**^B^**	56
E7.0	16 (23.18)	40 (57.97)	13 (18.84)	69
E6.5	9 (23.68)	21 (55.26)	8 (21.05)	38

Chi-square test: A; p = 0.0015, B; p = 0.0261.

To determine whether the early lethality of *Meteorin^−/−^* embryos is due to abnormality prior to implantation, we collected E3.5 blastocysts from heterozygote intercrosses and cultured them individually for 5 days. Two days after culture, all blastocysts had successfully attached to the bottom of the culture dish and hatched from the zona pellucida. Five days after culture, all the blastocysts displayed apparently normal outgrowth of trophoblast giant cells and inner cell mass (Fig. S2A–C). *In situ* hybridization of *Pou domain class 5 transcription factor 1* (*Pou5f1*) (also known as *Oct3/4*), a marker of inner cell mass also shows that there is no difference between wild-type and Meteorin-null embryos (Fig. S2D–G). Taken together, it suggests that *Meteorin^−/−^* embryos develop normally prior to implantation.

**Figure 2:**
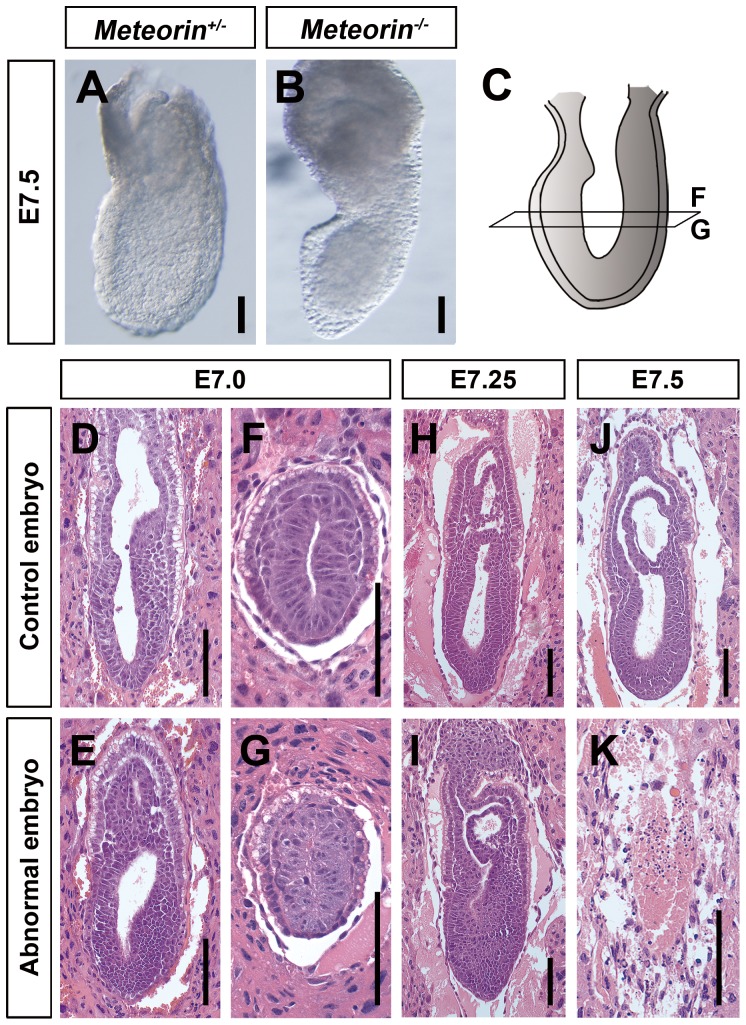
Developmental defects in *Meteorin^−/−^* embryos during gastrulation. **(A, B)** Gross morphology of *Meteorin^+/+^* and *Meteorin^−/−^* embryos at E7.5. A fraction of *Meteorin^−/−^* embryos survived at E7.5 and these were markedly smaller than *Meteorin^+/+^* embryos. **(C)** A schematic view indicates where the transverse section images of panels F and G were obtained. **(D–K)** Paraffin sections of each decidua from *Meteorin* heterozygous matings were stained with hematoxylin and eosin. At E7.0, slightly thickened cell layers were observed in the presumptive primitive streak in abnormal embryos **(E and I)** and this accumulation of cells became more apparent at E7.25 **(I)**. At E7.5, some embryos were resorbed and were no longer seen in deciduae **(K)**. All scale bars: 100 µm.

As blastocyst outgrowth experiments revealed normal *Meteorin^−/−^* embryo development prior to implantation, we examined whether there were developmental defects during the gastrulation stage in *Meteorin^−/−^* embryos. As shown in Fig. 2A and B, viable *Meteorin^−/−^* embryos at E7.5 were much smaller than controls, especially in the embryonic region, suggesting that Meteorin may play a role in embryonic development. Histological analysis showed that there was no apparent difference between embryos from intercrosses until E6.75 (data not shown). Mesendoderm cells derived from the epiblast are specified between E7.0 and E7.5 and migrate through the primitive streak [8]. Between E7.0 and E7.25, around one-fourth of embryos from heterozygous matings displayed abnormal accumulation of cells in the presumptive primitive streak toward the posterior or distal region of the embryos, suggesting that impaired mesendoderm development and disorganized embryonic integrity might lead to lethality and empty deciduae at E7.5 (Fig. 2D–K).

### Meteorin-null EB culture displays defective mesendodermal differentiation

**Figure 3:**
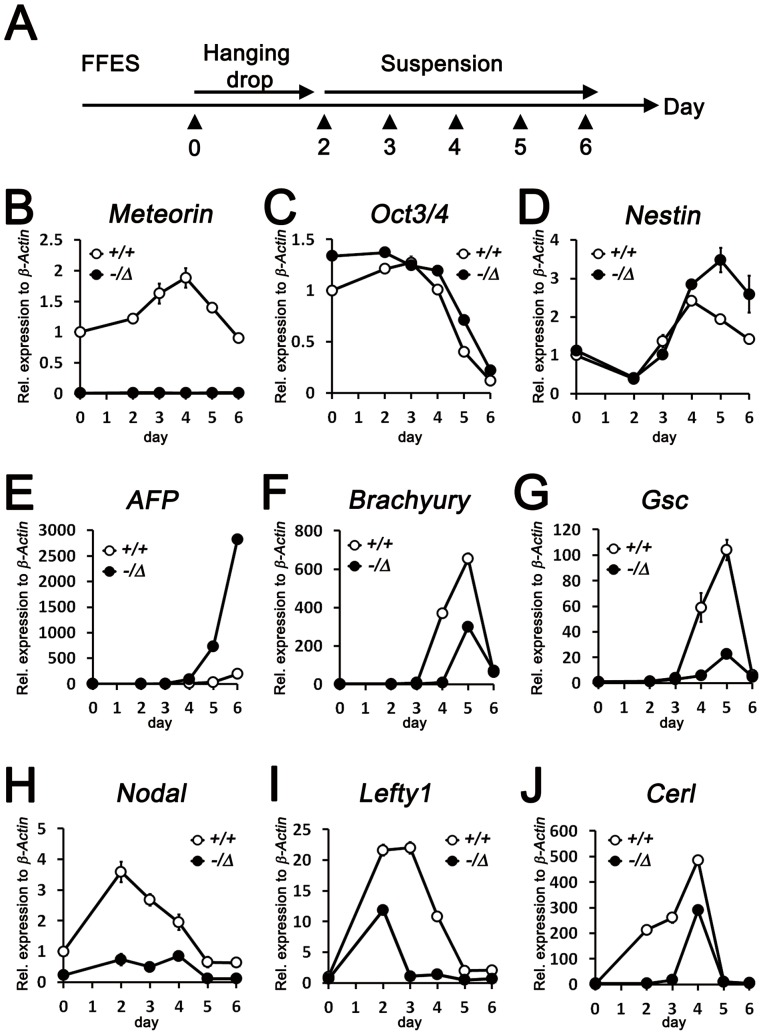
Impaired mesendodermal development in EB culture of *Meteorin^−/Δ^* cells. **(A)** Schematic view of EB culture. After 2 passages maintained in feeder-free embryonic stem cells, cells were dissociated and cultured using hanging drops for 2 days. Then, they were collected and cultured on bacterial-grade dishes. At each day of culture, indicated as arrowheads in the schematic view, cells were harvested for subsequent qRT-PCR experiments. **(B–H)** The expression of early developmental lineage markers on each day of culture was analyzed by qRT-PCR. **(B)** Successful gene deletion in *Meteorin^−/Δ^* ES cells was confirmed by observing the transcript level of *Meteorin*. **(C)** Expression of *Oct3/4*, a pluripotency marker, was similarly maintained in *Meteorin^−/Δ^* EB culture compared to *Meteorin^+/+^* EB culture. Expression levels of *Nestin*, an early neuroectoderm marker **(D)**, and *AFP*, a visceral endoderm marker **(E)**, were higher in *Meteorin^−/Δ^* EB culture than in *Meteorin^+/+^* EB culture. The expression levels of *Brachyury*, an early mesendoderm marker **(F)**, *Gsc*, an endoderm marker **(G)**, and *Nodal*, a posterior epiblast marker **(H)**, significantly decreased in *Meteorin^−/Δ^* EB culture throughout the culture period. The expression of *Lefty1* **(I)** and *Cerl* **(J)**, downstream molecules of Nodal signaling, was also lower in *Meteorin^−/Δ^* EB culture than in the control. Error bars indicate standard error of the mean (s.e.m.). All experiments were conducted more than 3 times and the representative graphs are shown.

To investigate the nature of *Meteorin^−/−^* embryo abnormalities, we adopted the *in vitro* EB culture system, which recapitulates the differentiation process during gastrulation. To first generate Meteorin-null ES cells, we sequentially targeted both alleles of the *Meteorin* gene by homologous recombination (Fig. S3A and B). After the forced expression of Cre recombinase, *Meteorin* transcripts were no longer expressed in *Meteorin^−/Δ^* ES cells (Fig. S3D and Fig. 3B). There was no apparent difference in morphology or proliferation rate between *Meteorin^−/Δ^* and control ES cells (data not shown).

Using these ES cells, we performed EB culture experiments. When ES cells are grown in medium with leukemia inhibitory factor (LIF), they can proliferate indefinitely while retaining their self-renewing activity. However, upon the removal of LIF, differentiation into three germ layers, the endoderm, mesoderm, and ectoderm, occurs spontaneously as in embryonic development [30]. After two days in hanging drop culture, cells were re-plated on bacterial-grade dishes in suspension for 4 consecutive days, and mRNA was harvested on each day of culture for subsequent qRT-PCR experiments (Fig. 3A). As EBs developed, the expression of the pluripotency markers, *Oct3/4* and *Nanog*, gradually decreased in the control EB culture, while the expression of the differentiation markers *Nestin, Neurofilament-M* (*NFM*), *Alpha-fetoprotein* (*AFP*), *Transthyretin* (*Ttr*), *Brachyury* (*T*), *Fetal liver kinase 1* (*Flk1*), *Goosecoid* (*Gsc*), and *Mix1 homeobox-like 1 (Mixl1)* was elevated (Fig. 3C–G and Fig. S4A–E) as reported earlier [31]. In the *Meteorin^−/Δ^* EB culture, the *Oct3/4* and *Nanog* levels were comparable to those of the controls, suggesting that pluripotency is not influenced by *Meteorin* disruption (Fig. 3C and Fig. S4A). However, the expression of neuroectoderm markers, *Nestin and NFM*, and visceral endoderm markers, *AFP and Ttr* was significantly higher in the *Meteorin^−/Δ^* EB culture than in the controls (Fig. 3D–E and Fig. S4B–C). In contrast, the expression of mesoderm markers, *Brachyury* and *Flk1*, and definitive endoderm markers, *Gsc* and *Mixl1*, was significantly lower in *Meteorin^−/Δ^* EB culture than in the controls (Fig. 3F–G and Fig. S4D–E), suggesting that the differentiation potential toward the mesendoderm is specifically affected by the disruption of *Meteorin*.

### Reduced Nodal signaling in Meteorin-null ES cells and embryos

When Nodal signaling, an important event in early embryonic patterning, is inhibited, mesendoderm formation is abrogated, while epiblast cells precociously differentiate toward the neuroectoderm [7], [16], [32]. To elucidate whether the defective differentiation in the *Meteorin^−/Δ^* EB culture is due to impaired Nodal signaling, we examined the expression of *Nodal* and its downstream genes, *Lefty1* and *Cerl*. Intriguingly, all of these molecules were significantly downregulated in the *Meteorin^−/Δ^* EB culture compared to the controls (Fig. 3H–J), suggesting that the defects in mesendoderm differentiation in the *Meteorin^−/Δ^* EB culture are a result of decreased Nodal signaling.

**Figure 4:**
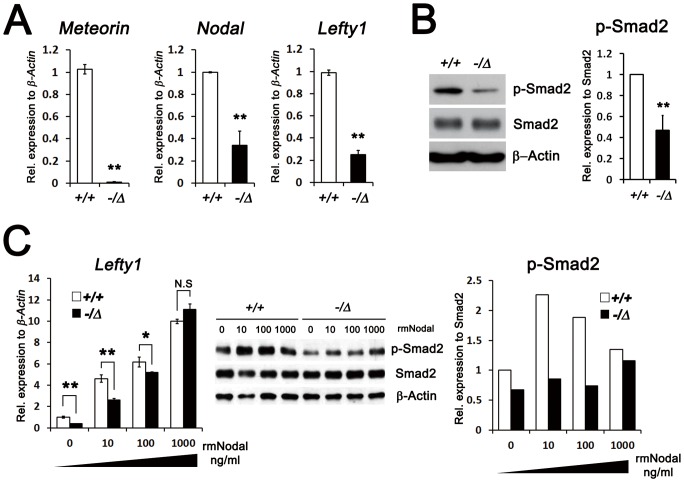
Reduced, but intact, Nodal signal transduction pathway in *Meteorin^−/Δ^* ES cells. **(A)** Relative levels of *Meteorin*, *Nodal*, and *Lefty1* in *Meteorin^+/+^* and *Meteorin^−/Δ^* ES cells were analyzed by qRT-PCR. In *Meteorin^−/Δ^* ES cells, the transcript levels of these genes decreased. Error bars indicate standard deviation (s.d.). **p<0.01. **(B)** Smad2 and its phosphorylated level (p-Smad2) were investigated by western blotting, and the protein loading was normalized to β-Actin. In *Meteorin^−/Δ^* ES cells, the phosphorylated level of Smad2 significantly decreased. Scanned blot images were measured by Scion image. Error bars indicate s.d. **p<0.01. **(C)** Expression of *Lefty1*, a downstream molecule of Nodal signaling, was assessed by qRT-PCR at 24 hours after addition of recombinant mouse Nodal (rmNodal) protein to *Meteorin^+/+^* or *Meteorin^−/Δ^* ES cells. Levels of Smad2 and p-Smad2 were also measured by western blotting at 2 hours after rmNodal treatment. The reduced levels of *Lefty1* transcript and p-Smad2 were rescued by rmNodal treatment in *Meteorin^−/Δ^* ES cells. Scanned blot images were measured by Scion image.

To investigate whether the decreased Nodal signaling is caused by reduced expression of *Nodal* in the *Meteorin^−/Δ^* ES cells themselves, we assessed the expression of *Nodal* and *Lefty1* and found that it was lower in *Meteorin^−/Δ^* ES cells than in the controls (Fig. 4A). In vertebrates, Nodal signaling is transmitted through Smad2 phosphorylation and its subsequent translocation into the nucleus acting as a transcription factor [16]. Indeed, the phosphorylated level of Smad2 in *Meteorin^−/Δ^* ES cells was significantly lower than that of the control ES cells (Fig. 4B). To examine whether *Meteorin^−/Δ^* ES cells show any defect in the Nodal signaling pathway itself, we stimulated *Meteorin^−/Δ^* ES cells with recombinant mouse Nodal (rmNodal) protein. The expression of *Lefty1* and the phosphorylation of Smad2 were rescued by the addition of exogenous rmNodal in *Meteorin^−/Δ^* ES cells (Fig. 4C), indicating that the Nodal signaling pathway remains intact in *Meteorin^−/Δ^* ES cells and that the reduced expression of *Nodal* itself might be regulated by Meteorin.

**Figure 5:**
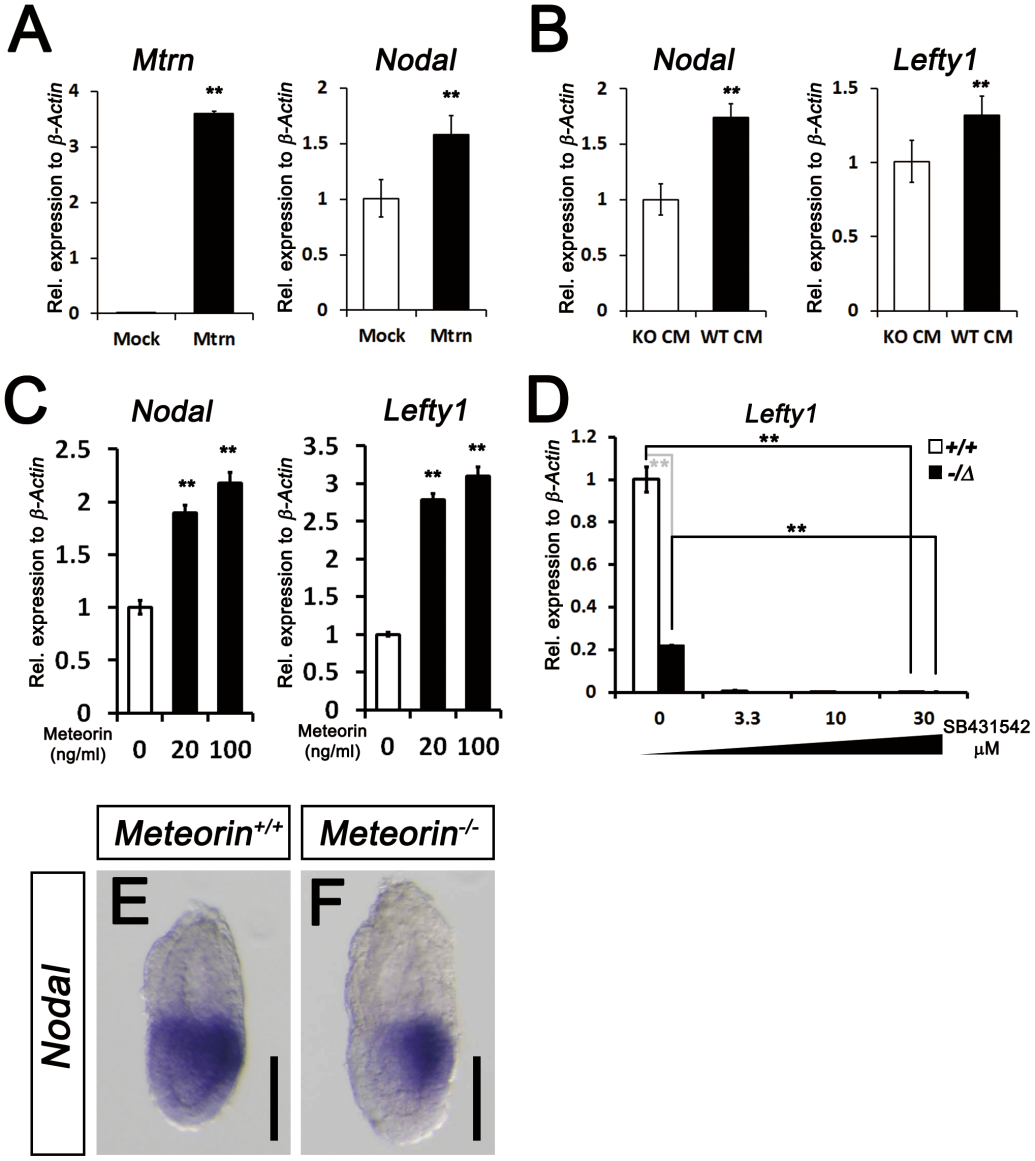
Restoration of Nodal signaling in *Meteorin^+/Δ^* ES cells by Meteorin expression and reduced expression of *Nodal* in *Meteorin^−/−^* embryos. **(A)** At 48-*mMeteorin* plasmid into *Meteorin^−/Δ^* ES cells, *Meteorin* and *Nodal* expression was higher than that of pCAGGS mock-transfected *Meteorin^−/Δ^* ES cells. **(B)** When conditioned medium obtained from *Meteorin^+/+^* ES cells (WT CM) was applied to *Meteorin^−/Δ^* ES cells, the expression levels of *Nodal* and *Lefty1* were higher than those seen when conditioned medium from *Meteorin^−/Δ^* (KO CM) was applied. **(C)** *Nodal* and *Lefty1* expressions were analyzed by qRT-PCR 48 hrs after the addition of recombinant mouse Meteorin (rmMeteorin) to *Meteorin^−/Δ^* ES cells. Both gene expressions were induced by Meteorin protein addition. Error bars indicate s.e.m. **p<0.01. **(D)** Expression of *Lefty1* transcript was assessed by qRT-PCR after the addition of SB431542, an inhibitor of type I TGF-beta receptors, to *Meteorin^+/+^* or *Meteorin^−/Δ^* ES cell lines. The residual activity of TGF-beta/Nodal signaling in *Meteorin^−/Δ^* ES cells was further inhibited by SB431542 treatment. Error bars indicate s.e.m. **p<0.01. **(E–F)** Expression of *Nodal* transcripts was analyzed by *in situ* hybridization in *Meteorin^+/+^* and *Meteorin^−/−^* embryos at each developmental stage. At E6.5, the expression level of *Nodal* was significantly lower in *Meteorin^−/−^* embryos than in *Meteorin^+/+^* embryos, especially in the proximal epiblast. All scale bars: 100 µm.

We next examined whether reduced *Nodal* expression in the *Meteorin^−/Δ^* ES cells can be rescued by introduction of Meteorin. When *Meteorin^−/Δ^* ES cells were transfected with a Meteorin-expressing vector, the expression of *Nodal* increased (Fig. 5A). Since Meteorin is a secreted protein, we applied the conditioned medium (CM) obtained from control ES cells to *Meteorin^−/Δ^* ES cells. As expected, the addition of control CM to *Meteorin^−/Δ^* ES cells led to increase in *Nodal* and *Lefty1* expression (Fig. 5B). To rule out the possibility that this result was caused by Nodal protein in the conditioned medium, not by Meteorin protein, we directly applied recombinant mouse Meteorin (rmMeteorin) protein to *Meteorin^−/Δ^* ES cells. The addition of rmMeteorin to Meteorin-deficient ES cells also rescued the expression of *Nodal* and *Lefty1*, directly suggesting that Meteorin is required to produce sufficient amounts of Nodal signaling for normal development (Fig. 5C). On the other hand, residual *Lefty1* expression in the *Meteorin^−/Δ^* ES cells was further decreased by SB431542, an inhibitor of type I TGF-beta receptors, (Fig. 5D), indicating that TGF-beta/Nodal signaling in the *Meteorin^−/Δ^* ES cells, albeit decreased, is functional. Indeed, in *Meteorin^−/−^* embryos, *Nodal* expresses, but the level was relatively lower than that in *Meteorin^+/+^* embryos, especially in the proximal region of embryos (Fig. 5E–F).

### The expression of endoderm-specific markers is severely affected in *Meteorin^−/−^* embryos

**Figure 6:**
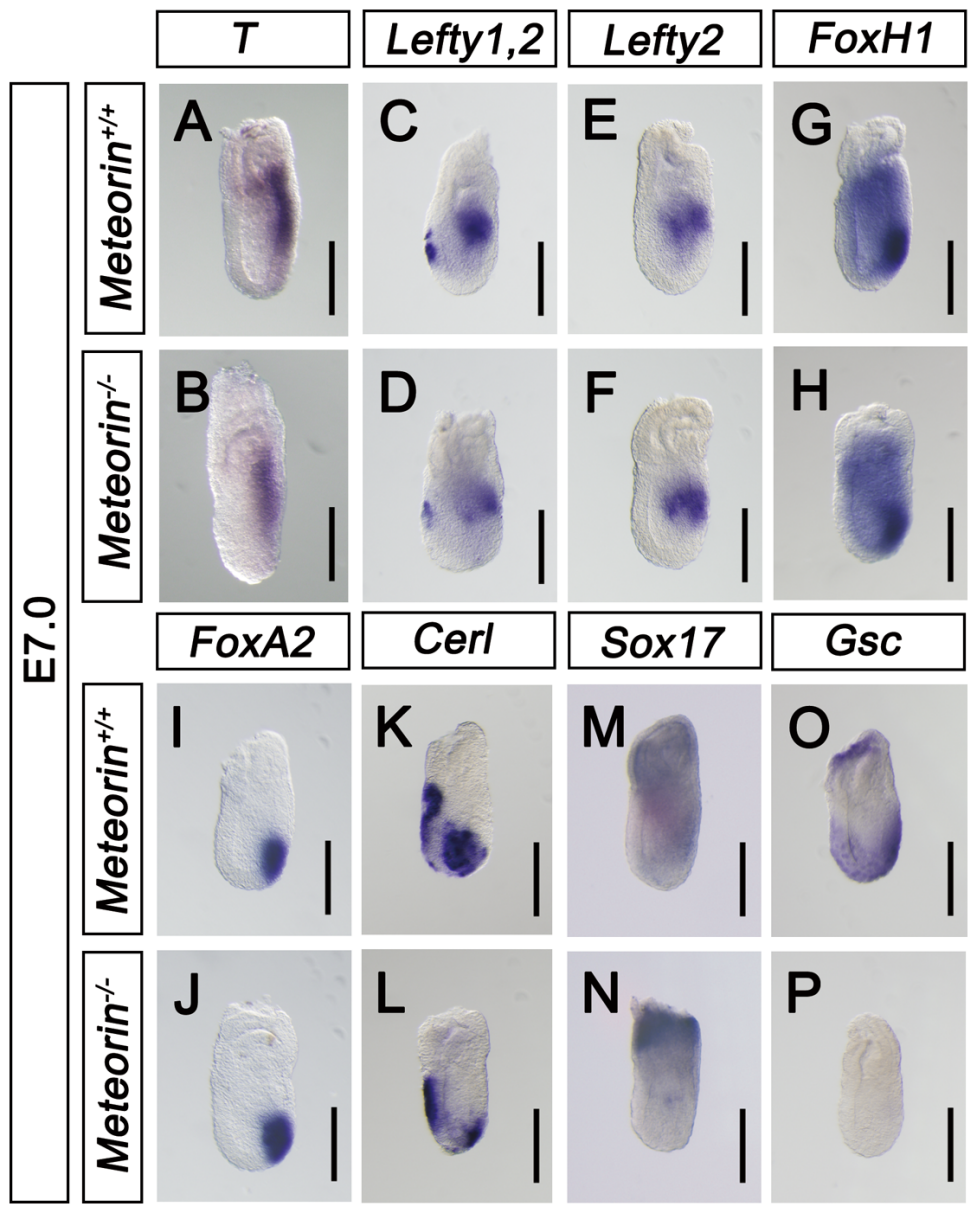
Selective reduction of the early endoderm markers *Cerl* and *Gsc* in Meteorin*^−^* ^/*−*^ embryos. At E7.0, the expression of various developmental markers and Nodal signaling downstream molecules was analyzed by *in situ* hybridization with each specific probe in Meteorin^+/+^ and Meteorin^−/−^ embryos. **(A and B)** *Brachyury (T)* expression levels were comparable in *Meteorin^−/−^ and Meteorin^+/+^* embryos. **(C–F)** The expression levels of *Lefty1* and *Lefty2*, downstream molecules of Nodal signaling, were analyzed. The expression levels of *Lefty1* in the anterior visceral endoderm and *Lefty2* in the posterior primitive streak in *Meteorin^−/−^* embryos were similar to those of control embryos. **(G and H)** The expression level of *FoxH1*, a transcriptional coregulator of Nodal, was not changed in *Meteorin^−/−^* embryos compared to controls. **(I and J)** Both the expression domain and the level of *FoxA2*, a marker of the anterior primitive streak, were maintained in *Meteorin^−/−^* embryos. **(K and L)** Expression of *Cerl*, a marker of the anterior visceral endoderm and definitive endoderm, was downregulated specifically in the anterior primitive streak region of *Meteorin^−/−^* embryos compared to that of control embryos. **(M and N)** *Sox17* was expressed in extra-embryonic ectoderm and definitive endoderm, and its expression in definitive endoderm was markedly reduced in *Meteorin^−/−^* embryos compared to controls. **(O and P)** Also, the expression of *Gsc*, another marker of definitive endoderm, was completely absent in *Meteorin^−/−^* embryos. For all experiments, at least three samples for each genotype were analyzed. All scale bars: 200 µm.

When Nodal signaling is completely blocked, the entire primitive streak is not formed [15], [16]. However, in mouse genetic models in which Nodal signaling is reduced, mesendoderm specification is selectively compromised, whereas primitive streak formation is preserved [7], [26], [33], [34]. Since *Nodal* transcripts were downregulated in *Meteorin^−/−^* embryos, we investigated whether the embryos displayed the developmental abnormalities frequently observed in Nodal signaling-compromised mutants [7], [26], [33]. As expected, the expression of *Brachyury (T)* and *BMP4* in the *Meteorin^−/−^* embryos was comparable to that in wild-type embryos (Fig. 6A–B and Fig. S5A–B). *Meteorin^−/−^* embryos also showed comparable expression of both *Lefty1* and *Lefty2* in the anterior visceral endoderm and posterior primitive streak, respectively (Fig. 6C–F). In addition, *FoxH1* and *FoxA2*, anterior primitive streak markers, were expressed in *Meteorin^−/−^* embryos (Fig. 6G–J), suggesting that mesodermal, extra-embryonic ectodermal, and visceral endodermal development occurred properly in the absence of Meteorin.

*Cerl* is expressed in the anterior visceral endoderm and definitive endoderm in the mid-gastrulation stage [5] (Fig. 6K). While the expression of *Cerl* in the anterior visceral endoderm is maintained in *Meteorin^−/−^* embryos, its expression in the definitive endoderm was specifically reduced (Fig. 6K and L). A marker of definitive endoderm, *Sox17* was also down-regulated in *Meteorin^−/−^* embryos. (Fig. 6M and N). Consistently, the expression of *Gsc* was also absent in the *Meteorin^−/−^* embryos, suggesting that endoderm specification is selectively inhibited in *Meteorin^−/−^* embryos (Fig. 6O and P). From these data, we conclude that the specification of the definitive endoderm is blocked because of impaired Nodal signaling in *Meteorin^−/−^* embryos.

## Discussion

To date, several studies have suggested Meteorin as a secreted protein which might be implicated in neurogenesis, gliogenesis, and angiogenesis [20], [21], [22]. In this study, we unexpectedly found that disruption of the *Meteorin* gene resulted in early embryonic lethality around E7.5. Indeed, *in situ* analysis revealed that *Meteorin* is expressed in the inner cell mass of blastocysts even before implantation and further in the epiblast and extra-embryonic ectoderm throughout peri-gastrulation stages, suggesting that Meteorin should play a critical role in early embryonic development. The EB culture experiments using *Meteorin^−/Δ^* ES cells showed that differentiation into the mesendoderm is selectively inhibited due to decrease in Nodal signaling. Through further experiments using *Meteorin^−/Δ^* ES cells and *Meteorin^−/−^* embryos, we found that Meteorin regulates the expression of *Nodal*, which is required for definitive endoderm development during gastrulation.

Although previous studies showed that Nodal signaling is crucial for mesendoderm allocation [35], [36] and we found that Nodal signaling is inhibited in *Meteorin^−/Δ^*ES cells, we couldn't clearly address whether the reduced Nodal signaling is entirely responsible for all the defects in *Meteorin^−/Δ^* EB culture because we cannot impeccably mimic the Nodal signaling of control EB culture in Meteorin-deficient EB culture. Considering our results that the rmNodal addition to Meteorin-null ES cells rescues the expression of target gene of Nodal signaling and phosphorylation of Smad2, and the previous data from Takenaga *et al.* that the Nodal-overexpressing ES cells can generate mesoderm and endoderm cells at the expense of neuroectoderm cell [35], we believe that the decline in Nodal signaling in Meteorin-deficient ES cells is responsible for the reduction of the mesendoderm formation and the increase of the neuroectoderm lineage cells. However, we cannot completely rule out the possibility of existence of other Nodal-independent signaling pathways.

Nodal is initially expressed throughout the epiblast prior to gastrulation [15], which is required for the induction of distal visceral endoderm expression of *Cerl* and *Lefty1* at the distal tip of the embryo [10], [12] as well as the movement of this visceral endoderm toward the anterior part of the embryo [11]. Cerl and Lefty1 act as negative regulators of Nodal to generate a gradient of Nodal signaling along the A–P axis, restricting the formation of the primitive streak to the posterior part of the embryo [5], [11]. Although Nodal signaling before gastrulation is important for A–P axis and proper primitive streak formation at the posterior region of the embryo, the development of these regions occurred properly in *Meteorin^−/−^* embryos, as *Cerl* and *Lefty1* were well expressed in the anterior visceral endoderm and *Brachyury* in the posterior region, respectively. Indeed, the expression of *Nodal* was not completely blocked in Meteorin-null embryos, as evident by *in situ* hybridization at E6.5, and *in vitro* experiments also showed that SB431542, an inhibitor of TGF-beta/Nodal signaling, further inhibited the expression of a downstream target of Nodal signaling in *Meteorin^−/Δ^* ES cells, suggesting that the remaining signaling activity of Nodal is sufficient for proper A–P axis and primitive streak formation.

As the primitive streak elongates to the distal tip of embryos, extra-embryonic mesoderm first arises from the posterior primitive streak, followed by the paraxial and lateral plate mesoderm in the middle and finally the anterior primitive streak derivatives, including axial mesoderm and definitive endoderm in the anterior-most region of the primitive streak [37]. Recent studies have demonstrated that the level of Nodal signaling is important for these cell lineage allocations during gastrulation. *Smad2^+/−^/Smad3^−/−^* and *Sox2Cre/Smad2^Robm1/CA^* embryos, in which Nodal signaling is reduced, exhibited formation of the primitive streak, but not of anterior primitive streak derivatives such as the definitive endoderm and prechordal plate [26], [33]. Nodal hypomorph mutant *Nodal^fl/Δ^* and *Nodal^ΔPEE/413.d^* embryos also showed defects in definitive endoderm and prechordal plate formation because of the decreased level of Nodal signaling [33], [34]. *Nodal^nr/nr^* mice, in which the mutant protein Nr cannot be potentiated by subtilisin-like proprotein convertases, also displayed selective mesendoderm defects due to reduced Nodal signaling activity [7]. Studies through genetic models in which Nodal signaling strength is abrogated to varying degrees suggest a model for mesendoderm allocation by Nodal signaling strength that the signaling strength governs the discrete cell fate decisions along the primitive streak. Specification into anterior primitive streak derivatives requires higher Nodal signaling, while lower levels of Nodal signaling are sufficient for the generation of paraxial and lateral plate mesoderm [7], [26]. In *Meteorin^−/−^* embryos, the expression of *Gsc, Sox17* and *Cerl*, markers of the definitive endoderm, was specifically downregulated at the anterior primitive streak, whereas the mesoderm was well specified, as evident by the expression of *Brachyury* in the elongating primitive streak possibly due to the reduced expression level of *Nodal* in epiblast. Thus, our data suggest that Meteorin is essential for Nodal expression levels to reach the threshold required to secure endodermal cell allocation.

In *Xenopus laevis* and *Zebrafish*, induction into the mesoderm and endoderm is dependent upon the strength of Nodal signaling. Low levels of Nodal signaling are sufficient for the expression of mesoderm markers, while higher levels induce endoderm marker expression [38], [39], [40], [41], [42]. Although it is not clear whether the discrete region of graded Nodal signaling is established during primitive streak elongation and how a subset of cells ingressing at the primitive streak attains increased Nodal signaling, it seems that the requirement of dose-dependent Nodal signaling for mesendoderm allocation is well conserved among species. To date, there is no report about Meteorin homologs in other species, but it seems that there are homologs of Meteorin in human, rat, and zebrafish based on sequence homology. Thus, further studies on whether the regulation of Nodal signaling by Meteorin is conserved among species would broaden our knowledge on how distinct lineage allocation is regulated during vertebrate development.

## Supporting Information

Figure S1 **Generation of Meteorin**-**null mice.** **(A)** Schematic view of the targeting strategy. Black boxes indicate exons and an open arrow depicts *diphtheria toxin A* (DTA). The position of the flanking probe is indicated by a grey dash and the expected fragment sizes after *Bam*HI digestion for Southern blotting are also provided. **(B)** Southern blotting of the *Bam*HI-digested gDNA derived from *Meteorin^+/+^* and *Meteorin^+/−^* mice. The flanking probe detects a 7.6-kb band for the wild-type allele (*+*) and an 11.1-kb band for the null allele (*-*). **(C)** Genotyping of blastocysts was conducted using primers corresponding to wild-type and null alleles. D.W: distilled water, S.M: size marker.
(TIF)

Figure S2 **Normal development of** ***Meteorin^−/−^*** **embryos until the blastocyst stage.** **(A–C)** Blastocysts obtained from heterozygous matings were cultured on gelatin-coated dishes for 5 days. All cells were lysed and genotyped after culture images were taken. All the blastocysts hatched normally. Inner cell mass aggregations on large, flat, and polyploidy trophoblast cells and scattered extra-embryonic endoderm cells were grown from each of blastocysts. **(D–E)** Expression of *Oct3/4*, an inner cell mass marker, was analyzed by *in situ* hybridization. Its expression in *Meteorin^−/−^* blastocyst was similar to that of *Meteorin^+/+^* blastocyst. All scale bars: 200 µm.
(TIF)

Figure S3 **Generation of** ***Meteorin^−/Δ^*** **ES cells through sequential targeting and Cre expression.** **(A)** Schematic of *Meteorin* targeting used for *Meteorin^−/Δ^* ES cell generation. The targeting construct was generated by flanking exons 3 and 4 with loxP sequences, and a neomycin-resistance cassette was used for subsequent selection of targeted ES cells. Black boxes indicate exons and an open arrow depicts DTA. **(B)** Schematic diagram of *Meteorin^−/Δ^* ES cell generation. **(C)** Southern blotting of the *Bam*HI-digested gDNA derived from ES cells at each targeting step. The flanking probe detected a 7.6-kb band from the wild-type allele (*+*), a 11.1-kb band from the null allele (−), an 8.9-kb band from the conditionally targeted allele (*f*), and a 7.1-kb band from the lox (*Δ*) allele obtained upon excision by Cre-recombinase. **(D)** *Meteorin* mRNA expression in each ES cell line was analyzed by qRT-PCR. In *Meteorin^+/f^* and *Meteorin^−/f^* ES cells, the level was reduced to half of that in *Meteorin^+/+^* ES cells, and no *Meteorin* expression was observed in *Meteorin^−/Δ^* ES cells.
(TIF)

Figure S4 **Defected mesendoderm development in EB culture of** ***Meteorin^−/Δ^*** **cells.** **(A–E)** Expression level of markers of several developmental lineages was analyzed by qRT-PCR. **(A)** Expression of *Nanog*, a pluripotency marker, was normal in *Meteorin^−/Δ^* EB culture compared to *Meteorin^+/+^* EB culture. Expression levels of *Neurofilament M* (*NFM*), an early neuroectoderm marker **(B)**, and *Transthyretin* (*Ttr*), a visceral endoderm marker **(C)**, were higher in *Meteorin^−/Δ^* EB culture than those in *Meteorin^+/+^* EB culture. The expression levels of *Fetal liver kinase 1 (Flk1)*, a mesoderm marker **(D)**, and *Mix1 homeobox-like 1 (Mixl1)*, an endoderm marker **(E)**, were significantly decreased in *Meteorin^−/Δ^* EB culture. Error bars indicate standard error of the mean (s.e.m.). All experiments were conducted more than 3 times and the representative graphs are shown.
(TIF)

Figure S5 **Normal development of extra-embryonic ectoderm in Meteorin-deficient embryos.** **(A–B)** *BMP4*, a marker for extra-embryonic ectoderm, expression is analyzed by *in situ* hybridization at E7.0. The expression of *BMP4* in *Meteorion^−/−^* embryos **(A)** was comparable with that of *Meteorin^+/+^* control embryos **(B)**.
(TIF)

Table S1 **Primer sets for qRT-PCR analysis and** ***in situ*** **hybridization probe cloning. **Sequences of primer sets used in this study are listed in the table.
(DOCX)
