# Supplementary figures and images for: Meteorin Regulates Mesendoderm Development by Enhancing Nodal Expression
Source: PLoS One. 2014 Feb 18;9(2):e88811. doi: 10.1371/journal.pone.0088811 (PMC3928293; doi:10.1371/journal.pone.0088811)

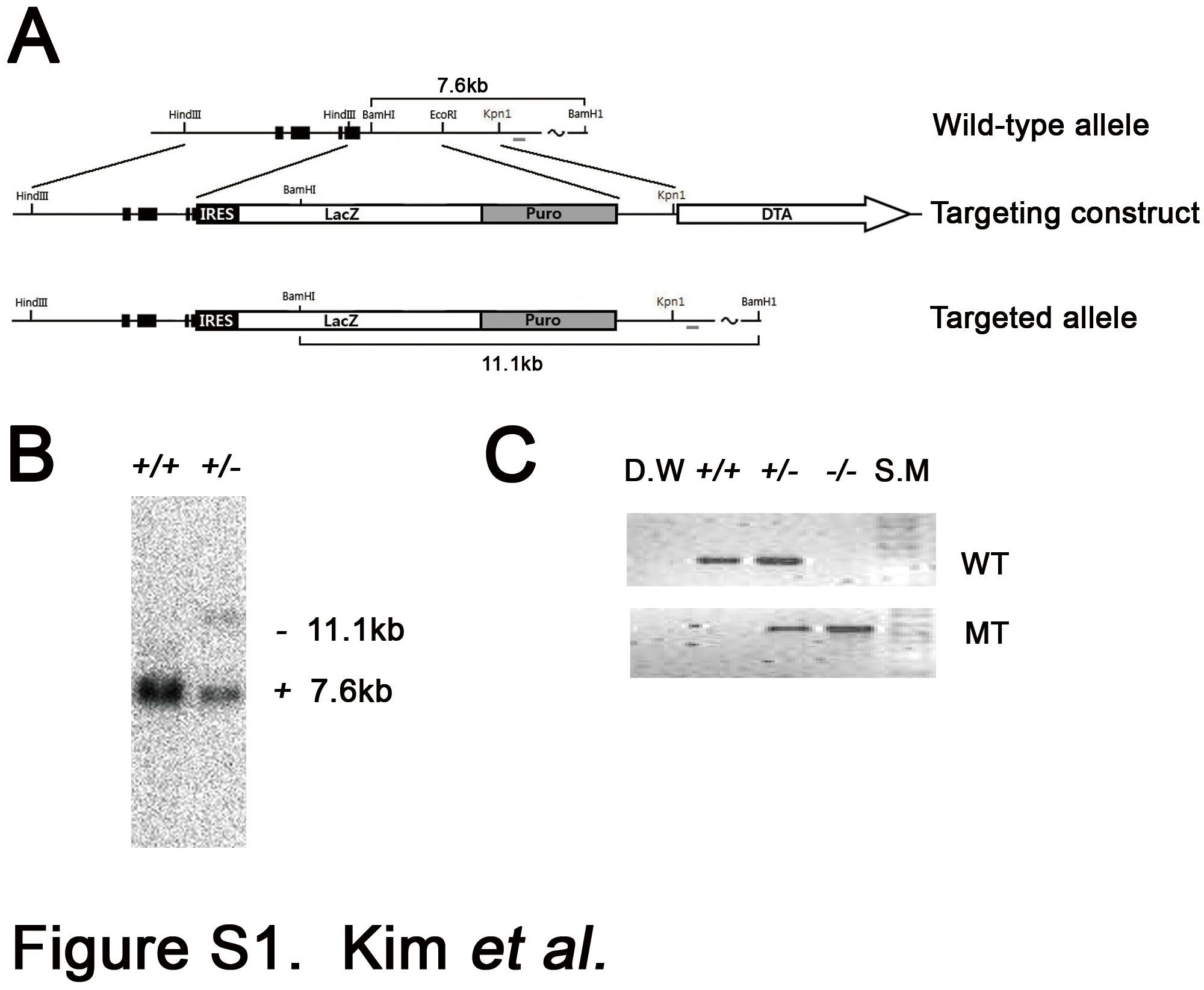

Supplement: Figure S1 — Generation of Meteorin-null mice. (A) Schematic view of the targeting strategy. Black boxes indicate exons and an open arrow depicts diphtheria toxin A (DTA). The position of the flanking probe is indicated by a grey dash and the expected fragment sizes after BamHI digestion for Southern blotting are also provided. (B) Southern blotting of the BamHI-digested gDNA derived from Meteorin+/+ and Meteorin+/− mice. The flanking probe detects a 7.6-kb band for the wild-type allele (+) and an 11.1-kb band for the null allele (-). (C) Genotyping of blastocysts was conducted using primers corresponding to wild-type and null alleles. D.W: distilled water, S.M: size marker. (TIF) [file pone.0088811.s001.tif]

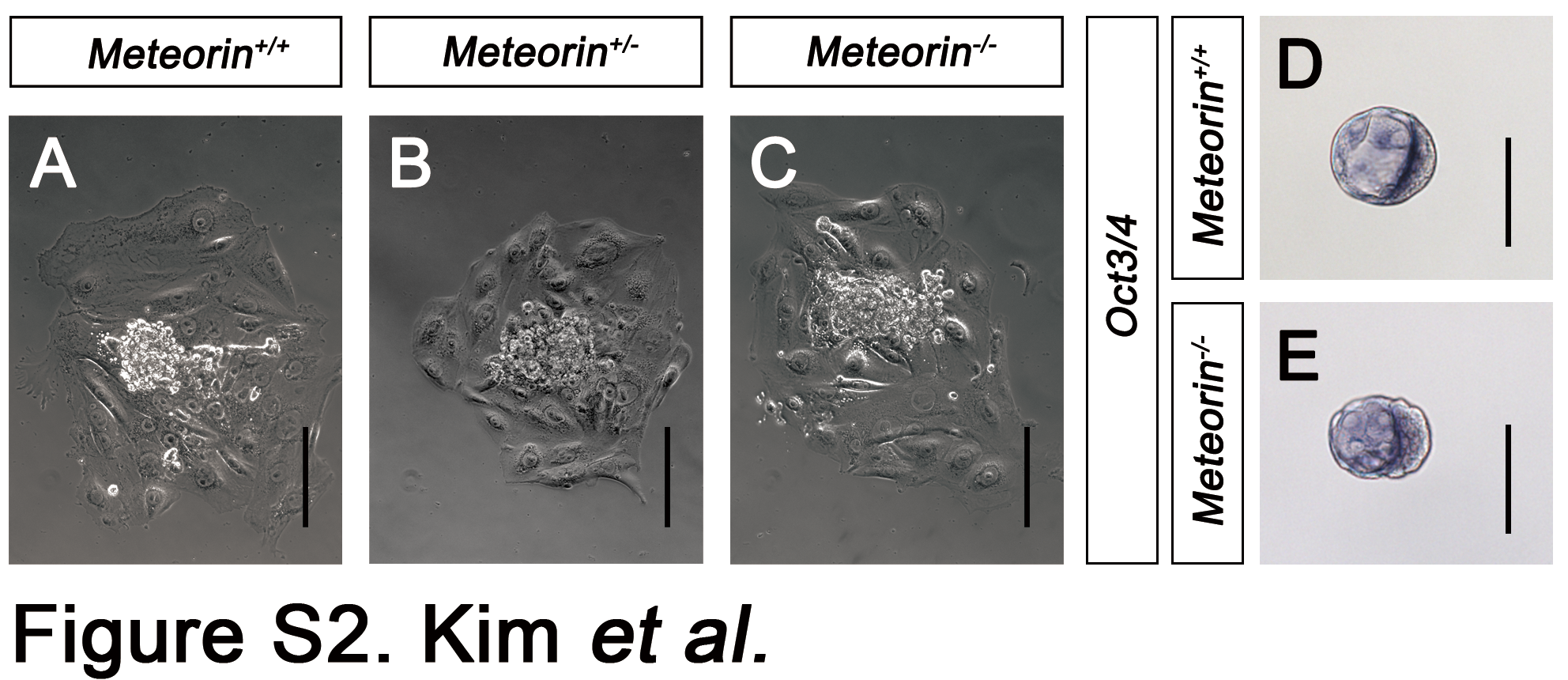

Supplement: Figure S2 — Normal development of Meteorin−/− embryos until the blastocyst stage. (A–C) Blastocysts obtained from heterozygous matings were cultured on gelatin-coated dishes for 5 days. All cells were lysed and genotyped after culture images were taken. All the blastocysts hatched normally. Inner cell mass aggregations on large, flat, and polyploidy trophoblast cells and scattered extra-embryonic endoderm cells were grown from each of blastocysts. (D–E) Expression of Oct3/4, an inner cell mass marker, was analyzed by in situ hybridization. Its expression in Meteorin−/− blastocyst was similar to that of Meteorin+/+ blastocyst. All scale bars: 200 µm. (TIF) [file pone.0088811.s002.tif]

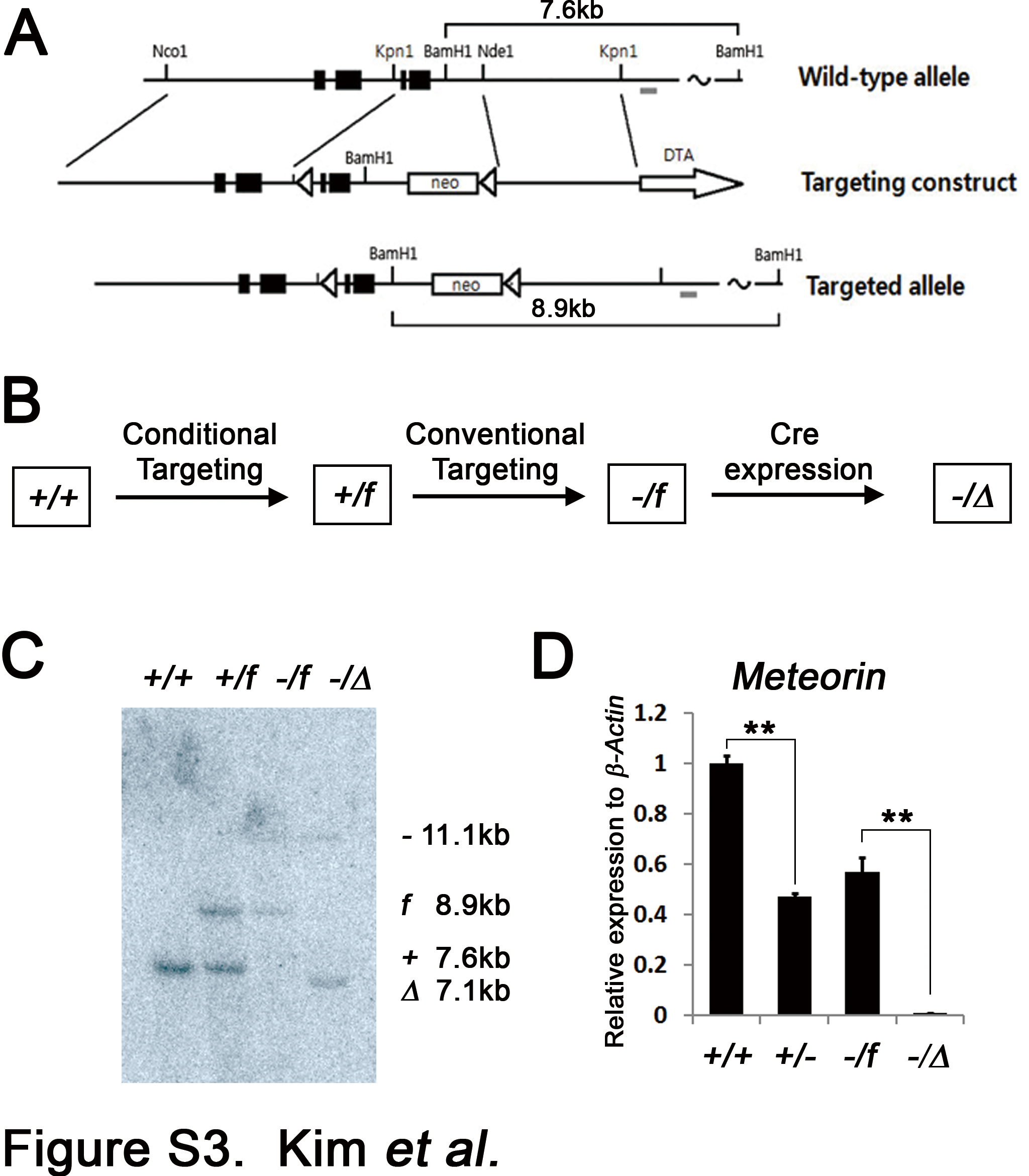

Supplement: Figure S3 — Generation of Meteorin−/Δ ES cells through sequential targeting and Cre expression. (A) Schematic of Meteorin targeting used for Meteorin−/Δ ES cell generation. The targeting construct was generated by flanking exons 3 and 4 with loxP sequences, and a neomycin-resistance cassette was used for subsequent selection of targeted ES cells. Black boxes indicate exons and an open arrow depicts DTA. (B) Schematic diagram of Meteorin−/Δ ES cell generation. (C) Southern blotting of the BamHI-digested gDNA derived from ES cells at each targeting step. The flanking probe detected a 7.6-kb band from the wild-type allele (+), a 11.1-kb band from the null allele (−), an 8.9-kb band from the conditionally targeted allele (f), and a 7.1-kb band from the lox (Δ) allele obtained upon excision by Cre-recombinase. (D) Meteorin mRNA expression in each ES cell line was analyzed by qRT-PCR. In Meteorin+/f and Meteorin−/f ES cells, the level was reduced to half of that in Meteorin+/+ ES cells, and no Meteorin expression was observed in Meteorin−/Δ ES cells. (TIF) [file pone.0088811.s003.tif]

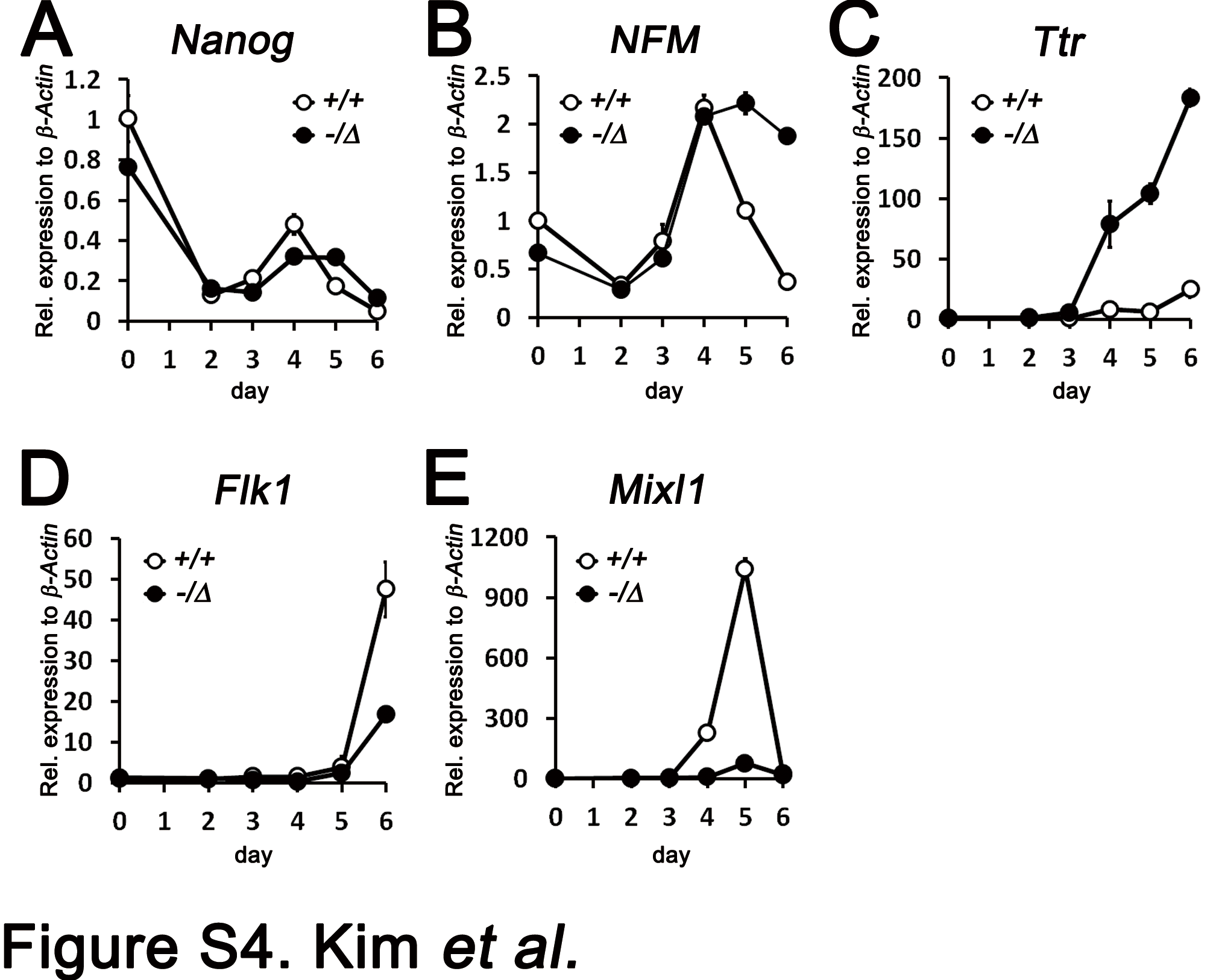

Supplement: Figure S4 — Defected mesendoderm development in EB culture of Meteorin−/Δ cells. (A–E) Expression level of markers of several developmental lineages was analyzed by qRT-PCR. (A) Expression of Nanog, a pluripotency marker, was normal in Meteorin−/Δ EB culture compared to Meteorin+/+ EB culture. Expression levels of Neurofilament M (NFM), an early neuroectoderm marker (B), and Transthyretin (Ttr), a visceral endoderm marker (C), were higher in Meteorin−/Δ EB culture than those in Meteorin+/+ EB culture. The expression levels of Fetal liver kinase 1 (Flk1), a mesoderm marker (D), and Mix1 homeobox-like 1 (Mixl1), an endoderm marker (E), were significantly decreased in Meteorin−/Δ EB culture. Error bars indicate standard error of the mean (s.e.m.). All experiments were conducted more than 3 times and the representative graphs are shown. (TIF) [file pone.0088811.s004.tif]

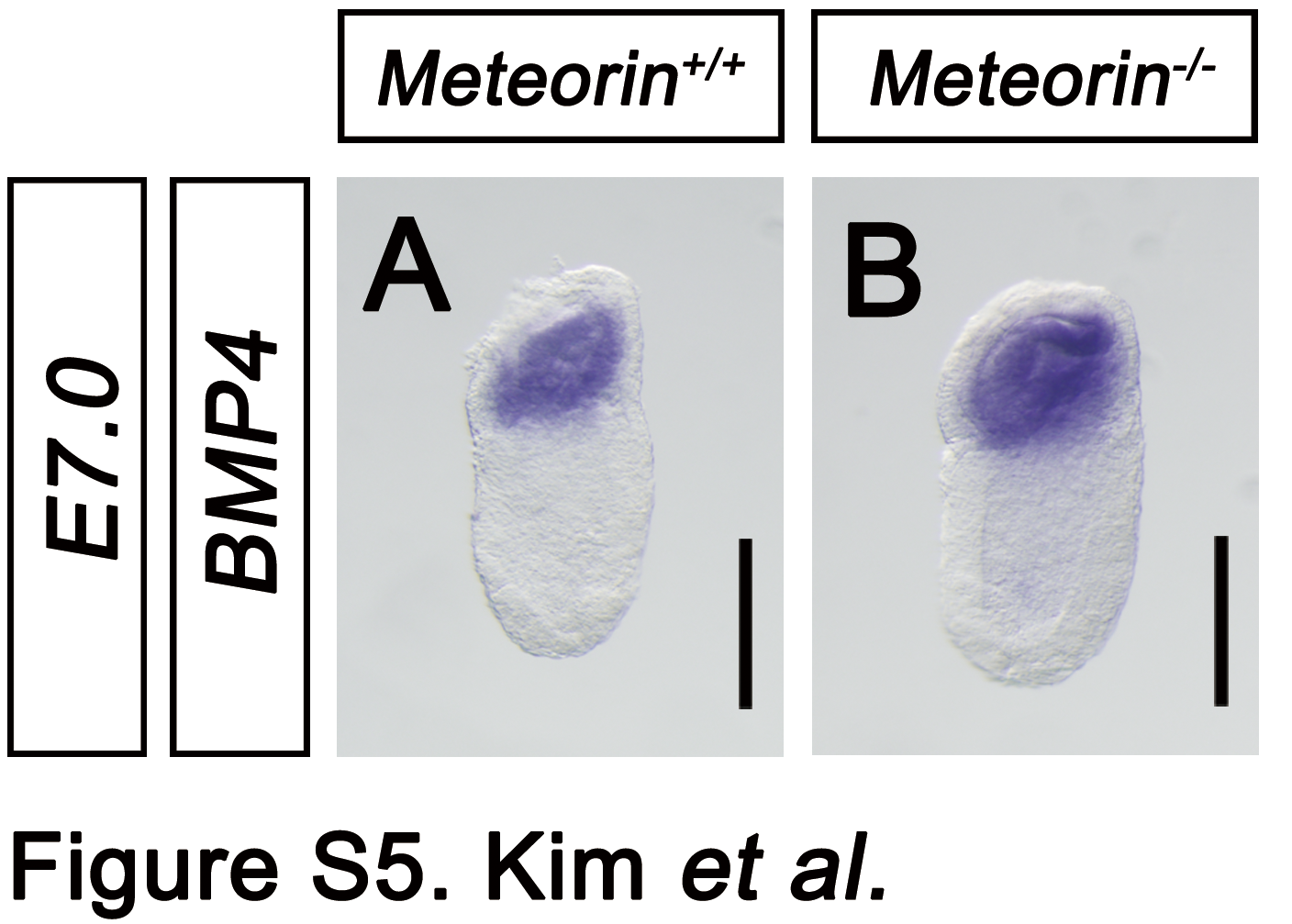

Supplement: Figure S5 — Normal development of extra-embryonic ectoderm in Meteorin-deficient embryos. (A–B) BMP4, a marker for extra-embryonic ectoderm, expression is analyzed by in situ hybridization at E7.0. The expression of BMP4 in Meteorion−/− embryos (A) was comparable with that of Meteorin+/+ control embryos (B). (TIF) [file pone.0088811.s005.tif]
