# Supplementary material for: Meteorin Regulates Mesendoderm Development by Enhancing Nodal Expression
Source: PLoS One. 2014 Feb 18;9(2):e88811. doi: 10.1371/journal.pone.0088811 (PMC3928293; doi:10.1371/journal.pone.0088811)
Supplement: Table S1 — Primer sets for qRT-PCR analysis and in situ hybridization probe cloning. Sequences of primer sets used in this study are listed in the table. (DOCX) [file pone.0088811.s006.docx]

| **Primer sets for qRT-PCR** | | | |
| --- | --- | --- | --- |
| **Genes** | **Forward primers** | **Reverse primers** | |
| ***β-Actin*** | 5ʹ-CCTAGAAGCACTTGCGGTGCACGATG-3ʹ | 5ʹ-TCATGAAGTGTGACGTTGACATCCGT-3ʹ | |
| ***Meteorin*** | 5ʹ-GCCTCACTTTGTCTACTCTCCA-3ʹ | 5ʹ-AACTACGGCACGGTTGCT-3ʹ | |
| ***Lefty1*** | 5ʹ-GCAGACTCAAGACCCTTTCA-3ʹ | 5ʹ-ACCATCCCTTCCACATCAG-3ʹ | |
| ***Lefty2*** | 5ʹ-GTGAGAACAGGGGAATCAAAA-3ʹ | 5ʹ-GGTTAGGAGAAATGGGAACAA-3ʹ | |
| ***Nodal*** | 5ʹ-ATCATTTGCCAGACAGAAGC-3ʹ | 5ʹ-GGTTGGTATCGTTTCAGCAG-3ʹ | |
| ***Goosecoid*** | 5ʹ-CGATTTGGACTCGGACAG-3ʹ | 5ʹ-GCTCCTCGTTGCTTTCTC-3ʹ | |
| ***Oct3/4*** | 5ʹ-CCACTTCACCACACTCTACTCA-3ʹ | 5ʹ-TCTCTTGTCTACCTCCCTTGC-3ʹ | |
| ***Nestin*** | 5ʹ-CCTCAACCCTCACCACTCTAT-3ʹ | 5ʹ-TGGCTGCTTCTTTCTTTACAA-3ʹ | |
| ***Brachyury*** | 5ʹ-CTCTTTCTTGCTGGACTTCG-3ʹ | 5ʹ-TGGTGAGTTTGACTTTGCT-3ʹ | |
| ***Alpha-fetoprotein*** | 5ʹ-TCACAGAAGAGGGTCCAAAGT-3ʹ | 5ʹ-AAAAGGCTCACACCAAAGAGT-3ʹ | |
| ***Cerl*** | 5ʹ-TTATCTGGCGTTGAAGAAGG-3ʹ | 5ʹ-TTGGAGACTTATGGCGTTTC-3ʹ | |
| ***Nanog*** | 5’-CCCTGATTCTTCTACCAGTCC-3’ | 5’-TCTGAAACCTGTCCTTGAGTG-3’ | |
| ***Neurofilament M*** | 5’-ATGAGGAGACCTTTGAGGAGA-3’ | 5’-GAACCCATTCTGTTTTGAAGC-3’ | |
| ***Ttr*** | 5’-CTGCTCCTAAAACCCAAAATC-3’ | 5’-TCCACTCTGCTTTCTGACCTA-3’ | |
| ***Flk1*** | 5’-TTTGGCAAATACAACCCTTCAGA-3’ | 5’-GCAGAAGATACTGTCACCACC-3’ | |
| ***Mixl1*** | 5’-ACGCAGTGCTTTCCAAACC-3’ | 5’-CCCGCAAGTGGATGTCTGG-3’ | |
| **Primer sets for *in situ* hybridization probe cloning** | | | |
| **Genes** | **Forward primers** | | **Reverse primers** |
| ***Lefty2*** | 5ʹ-TTGTTCCCATTTCTCCTAACC-3ʹ | | 5ʹ-TCTCTTGCCCCAGTCTTTTAT-3ʹ |
| ***FoxH1*** | 5ʹ- ACTATGAGGGCTGGAAGGAC-3ʹ | | 5ʹ-GGCAAGTAAGAAGTGGGTAGC-3ʹ |
| ***FoxA2*** | 5ʹ-TTCAACGACTGCTTCTCAAG-3ʹ | | 5ʹ-ATTTCCATTCCCTTCCCTATT-3ʹ |
| ***Goosecoid*** | 5ʹ-CACGGGGACTCGCTCTAC-3ʹ | | 5ʹ-TTGCCATCACTTTATTGTATTGTC-3ʹ |
| ***Nodal*** | 5ʹ-GCCACCATGAGTGCCCACAGCCTC-3ʹ | | 5ʹ-TGTCAGAGGCACCCACACTCCT-3ʹ |

**Supplementary Table 1.**
